# Glucagon changes substrate preference in gluconeogenesis

**DOI:** 10.1016/j.jbc.2022.102708

**Published:** 2022-11-17

**Authors:** Huiting Xu, Yujue Wang, Hyokjoon Kwon, Ankit Shah, Katarzyna Kalemba, Xiaoyang Su, Ling He, Fredric E. Wondisford

**Affiliations:** 1 Department of Medicine, Rutgers-Robert Wood Johnson Medical School, New Brunswick, New Jersey, USA; 2 Rutgers Cancer Institute of New Jersey, New Brunswick, New Jersey, USA; 3 Departments of Pediatrics and Pharmacology, Johns Hopkins University School of Medicine, Baltimore, Maryland, USA

**Keywords:** gluconeogenesis, glucagon, PKA, liver metabolism, glycerol, ATGL, adipose triglyceride lipase, DHAP, dihydroxyacetone phosphate, DM, diabetes mellitus, FFA, free fatty acid, GAP, glyceraldehyde 3-phosphate, GNG, gluconeogenesis, INSP3R1, inositol triphosphate receptor 1, MIDA, mass isotopomer distribution analysis, PCX, pyruvate carboxylase, PH, primary hepatocyte, PL, pyruvate and lactate, T2DM, type 2 diabetes mellitus, TCA, tricarboxylic acid, TPhos, triose-phosphate

## Abstract

Fasting hyperglycemia in diabetes mellitus is caused by unregulated glucagon secretion that activates gluconeogenesis (GNG) and increases the use of pyruvate, lactate, amino acids, and glycerol. Studies of GNG in hepatocytes, however, tend to test a limited number of substrates at nonphysiologic concentrations. Therefore, we treated cultured primary hepatocytes with three identical substrate mixtures of pyruvate/lactate, glutamine, and glycerol at serum fasting concentrations, where a different U-^13^C– or 2-^13^C–labeled substrate was substituted in each mix. In the absence of glucagon stimulation, 80% of the glucose produced in primary hepatocytes incorporated either one or two ^13^C-labeled glycerol molecules in a 1:1 ratio, reflecting the high overall activity of this pathway. In contrast, glucose produced from ^13^C-labeled pyruvate/lactate or glutamine rarely incorporated two labeled molecules. While glucagon increased the glycerol and pyruvate/lactate contributions to glucose carbon by 1.6- and 1.8-fold, respectively, the glutamine contribution to glucose carbon was increased 6.4-fold in primary hepatocytes. To account for substrate ^13^C carbon loss during metabolism, we also performed a metabolic flux analysis, which confirmed that the majority of glucose carbon produced by primary hepatocytes was from glycerol. *In vivo* studies using a PKA-activation mouse model that represents elevated glucagon activity confirmed that most circulating lactate carbons originated from glycerol, but very little glycerol was derived from lactate carbons, reflecting glycerol’s importance as a carbon donor to GNG. Given the diverse entry points for GNG substrates, hepatic glucagon action is unlikely to be due to a single mechanism.

Diabetes mellitus (DM) is currently a global health epidemic that affects nearly 10% of the population worldwide (1). In both type 1 DM and type 2 DM (T2DM), enhanced gluconeogenesis (GNG) is the major contributor to fasting hyperglycemia (2, 3, 4, 5). GNG takes place substantially in the liver, where three carbon substrates are converted to glucose (5). While defects in insulin signaling are central to diabetes pathogenesis, the effect of insulin signaling on GNG appears to be largely indirect through substrate availability (6, 7, 8, 9). Glucagon, in contrast, directly regulates glucose production by its action on hepatocytes (10, 11, 12). The secretion of glucagon is dependent on paracrine insulin signaling in the pancreatic islet, and the loss of paracrine regulation markedly increases glucagon levels in poorly controlled type 1 DM and in the late stage of T2DM (13, 14). Even in patients with early-stage T2DM where glucagon levels may not be elevated, glucagon levels are nonetheless inappropriate in the setting of hyperglycemia (15).

GNG is the anabolic process of making new glucose from substrates such as pyruvate, lactate, amino acids (AAs), and glycerol (5). There are three ways for carbons to enter GNG: (1) glycerol can be converted to glycerol-3-phosphate and enter the pathway as dihydroxyacetone phosphate (DHAP), (2) lactate and other 3-carbon AAs (*e.g.*, alanine) can be converted to pyruvate and enter the pathway as oxaloacetate, and (3) other gluconeogenic AAs (*e.g.*, glutamine) can first enter the tricarboxylic acid (TCA) cycle before being metabolized to oxaloacetate. At the early stage of fasting, glycogen catabolism in the muscle and other peripheral organs produces circulating lactate, which has long been assumed to be the dominant source of gluconeogenic carbon (3). However, extended fasting periods deplete glycogen stores, and lipolysis and proteolysis provide gluconeogenic carbon in the form of glycerol (Gro) and AAs, respectively (16). We have previously demonstrated that glycerol is an important gluconeogenic substrate in mouse primary hepatocytes (PHs) and *in vivo* (17). Furthermore, several clinical studies have emphasized the role of AAs and glycerol as substrates for GNG in diabetic patients (5, 18, 19, 20, 21, 22, 23, 24).

Glucagon is known to promote hepatic glucose production by activation of the glucagon receptor and G protein–coupled receptors (10, 25). A signaling cascade activated by glucagon ultimately leads to increased PKA activity, mediating increases in gluconeogenic enzymes such as glucose 6-phosphatase (G6PC) and phosphoenolpyruvate carboxykinase (PCK1) (26). However, a recent study suggests an alternative mechanism whereby glucagon enhances GNG by allosterically activating pyruvate carboxylase (PCX) through calcium-induced hepatic lipolysis and β-oxidation (8). Previous studies on glucose production in PHs and *in vivo* (8, 20) have generally not considered glycerol or have employed substrates at superphysiological concentrations to study glucagon’s effect. One *in vivo* study concluded that lactate has a dominant role in glucose metabolism and by extension GNG, given that lactate has very high turnover flux in mammals (27). However, given the many substrates that feed GNG, it is essential to evaluate multiple substrates to determine glucagon’s overall effect.

In this study, we utilized a novel ^13^C tracing method to study the effect of glucagon in the mouse PHs treated with physiological concentrations of substrates including glycerol, pyruvate, lactate, and glutamine and in mice treated with nonperturbative concentrations of these substrates. Glucagon increased glucose production from all four substrates in PHs, indicating PCX activity alone is insufficient to explain glucagon’s effect. Importantly, in the presence of all four substrates at physiological concentrations, 76% of GNG carbon originates from glycerol. Glucagon had three effects on substrate utilization in PHs, which are correlated with increased expression of G6PC and PCK1: (1) absolute GNG flux from all four substrates was increased; (2) glucagon preferentially increased glutamine use, and (3) relative contribution of glycerol is decreased although glycerol still remains the dominant GNG substrate in the absence or presence of glucagon stimulation. *In vivo* tracer infusion in PKA-activated mice confirm these findings.

## Results

### Glucagon-induced glucose production in PH

To recapitulate physiological fasting substrate concentration in glucagon-induced hepatic GNG, mouse PHs were cultured in physiological substrate concentrations found in mouse serum after a 12-h fast (28, 29). Under these *in vitro* conditions, issues of *in vivo* hepatic zonation of GNG and changes in substrate concentrations across vascular beds are eliminated. When cultured with individual substrates, glucagon significantly increased glucose production from each substrate, but glucose production was greatest when PH were cultured in the presence of all substrates (Fig. S1*A*). These media concentrations were maintained at a constant level over the 8-h period by supplementation (Fig. S1*B*).

**Figure 1:**
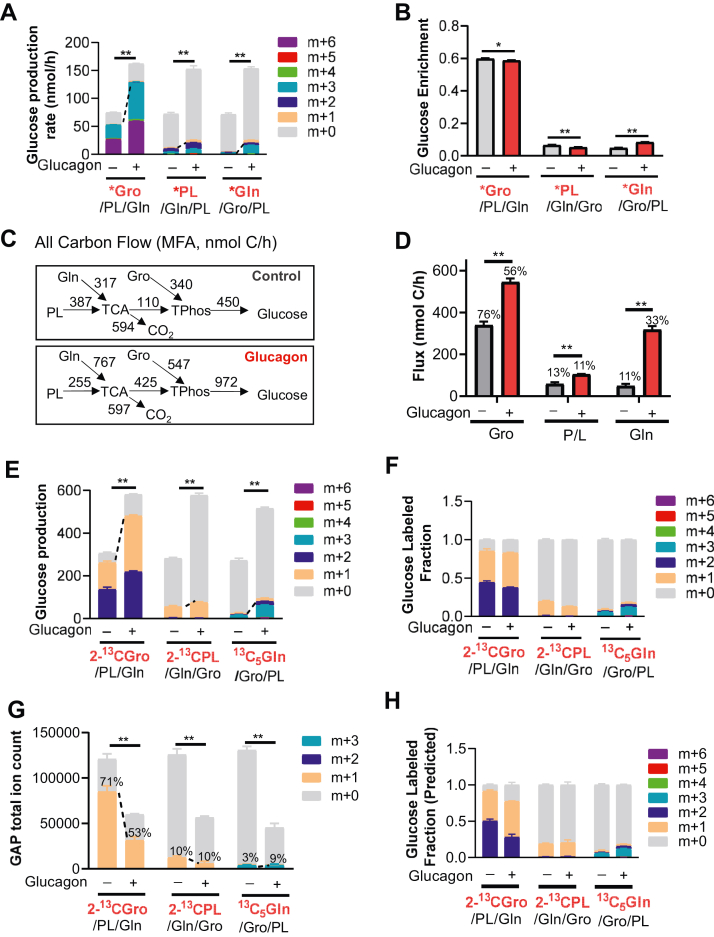
**Glycerol is the dominant substrate in mouse primary hepatocytes for glucagon-stimulated gluconeogenesis.***A*, labeling pattern of glucose production with or without treatment of 20 nM glucagon generated from four substrates labeled with U-^13^C tracers one at a time in mouse primary hepatocytes. The labeled substrates are shown in *red* in each group. *B*, glucose enrichment from glycerol, pyruvate/lactate, and glutamine with or without glucagon treatment in mouse primary hepatocytes. *C*, key flux measurements in mouse primary hepatocytes using an MFA analysis (nmol C/h). *D*, effective carbon flow to triose-phosphates from indicated substrates with or without glucagon stimulation. The distribution of carbon (based on flux) in the absence or presence of glucagon is shown as a percentage of the total. *E*, labeling pattern of glucose production in mouse primary hepatocytes after 8-h treatment of four substrates with 2-^13^C glycerol, 2-^13^C pyruvate/lactate, and U-^13^C glutamine, as indicated. *F*, glucose-labeled fraction in mouse primary hepatocytes treated with or without 20 nM glucagon in the four substrates with 2-^13^C glycerol, 2-^13^C pyruvate/lactate, and U-^13^C glutamine, as indicated. *G*, total ion count of GAP in mouse primary hepatocytes treated with or without 20 nM glucagon in the four substrates with 2-^13^C glycerol, 2-^13^C pyruvate/lactate, and U-^13^C glutamine, as indicated. *H*, predicted glucose-labeled fraction based on fractional GAP labeling using MIDA. For all graphs, SEM is shown, n = 9 biological replicates. Statistical analysis was performed using multiple t-tests between glucagon-treated and control groups. ∗*p* < 0.05, ∗∗*p* < 0.01. ^13^C isotopologues of glucose are indicated (m + 0 to m + 6). GAP, *glyceraldehyde 3-phosphate*; Gln, L-glutamine; MFA, metabolic flux analysis; MIDA, mass isotopomer distribution analysis.

To determine individual substrate contribution to GNG, we cultured mouse PHs in the same four-substrate mixture, substituting one of the substrates with a U-^13^C–labeled version (pyruvate and lactate mixture (PL) is considered as one substrate because of rapid interconversion). This results in three labeling schemes (Fig. 1*A*): ^13^C_3_ glycerol (∗Gro) with all other substrates unlabeled (∗Gro/PL/Gln), ^13^C_3_ PL (∗PL) with all other substrates unlabeled (Gro/∗PL/Gln), and ^13^C_5_ glutamine (∗Gln) with all other substrates unlabeled (Gro/PL/∗Gln). Since all substrates were tested at identical concentrations without or with ^13^C labeling, glucose production could be measured at nonperturbative concentrations and fractional labeling of glucose was maximized. Total glucose production rate in the media of all schemes was similar (Fig. 1*A*), indicating that the U-^13^C–labeled substrates did not alter the overall glucose production. Interestingly, however, 80% of the glucose produced after glucagon stimulation contained either one (m + 3) or two (m + 6) glycerol molecules. When all labeled substrates were considered, >95% of all glucose in the media was from GNG, indicating glucose released from glycogen into the media was minimal. ∗Gro/PL/Gln was the only substrate mix that generated m + 6 glucose (Fig. 1*A*), indicating the incorporation of two ^13^C_3_ glycerol molecules and increased efficiency of this pathway. In contrast, the predominant form of glucose synthesized from either ∗PL or ∗Gln was m + 3, followed by m + 2 and m + 1, the latter two species are due to ^13^C loss in the TCA cycle (Fig. 1*A*), which was confirmed using 2-^13^C–labeled substrates (see below). Average carbon enrichment was also lower with TCA-derived substrates due, in part, from ^13^C loss in the TCA cycle (note m + 1 and m + 2 species of ∗PL and ∗Gln, Fig. 1*B*).

### Shift of carbon source preference in glucagon-induced GNG

We next calculated the ^13^C flow from each substrate to glucose in mouse PHs using a previously validated *in vitro* model (Fig. 1, *C* and *D*, ref. (17)). Under basal conditions, the relative contribution to the triose-phosphate (TPhos; glyceraldehyde 3-phosphate (GAP) and DHAP) pool can be determined using the flux ratio of glycerol to TCA-derived substrates (pyruvate/lactate and glutamine). This ratio of 340/110 or 3.1-fold indicates that 76% of the carbons in glucose were derived from glycerol (Fig. 1, *C* and *D*). As expected, overall GNG flux increased approximately 2-fold after glucagon treatment (450–972 nmol C/h, Fig. 1*C*), but the flux ratio of glycerol to TCA-derived substrates dropped to 547/425 or 1.3-fold, indicating a preferential increase in TCA-derived substrate use. Further analysis suggests that relative glutamine flux to the TPhos pool was dramatically increased by glucagon (319/50 or 6.4-fold), while relative pyruvate/lactate flux to the TPhos pool was increased only 1.8-fold (106/60), similar to the 1.6-fold flux increase from glycerol after glucagon (547/340). These glucagon-induced changes reduced glycerol carbon contribution to glucose from 76% to 56% (Fig. 1*D*). Given that glucagon is thought to increase both glutamine and pyruvate/lactate flux (8, 20), we also determined the average ^13^C enrichment of gluconeogenic intermediates (Fig. S1*C*). After glucagon treatment, ^13^C enrichment of TCA intermediates was increased from glutamine but decreased from the pyruvate/lactate (Fig. S1*C*), consistent with the previous findings (20).

In PHs, U-^13^C pyruvate/lactate can be consumed in β-oxidation, leading to lower glucose labeling when free fatty acids (FFAs) are absent from the media. To test this hypothesis in PHs, we supplemented our four-substrate culture conditions with FFAs and found that FFA modestly increased total glucose production (Fig. S1*D*). However, FFA treatment had minimal or no effect on glucose production from pyruvate/lactate and glutamine. In fact, FFA supplementation reduced glucose production from glycerol after glucagon stimulation (Fig. S1*D*), likely due to higher cellular NADH levels from β-oxidation reducing glycerol flux through glycerol 3-phosphate dehydrogenase. Consistent with a recently described model (8), Fig. S2*E* demonstrates higher cellular acetyl-CoA levels after either glucagon or FFA treatment. Higher cellular NADH levels from β-oxidation would be predicted to reduce glycerol flux through glycerol-3-phosphate dehydrogenase and glutamine flux through the TCA cycle and might explain these results.

Using mixtures of uniformly ^13^C-labeled substrates, our data demonstrate that glycerol is most efficient at labeling the glucose synthesized in mouse PHS without any loss of ^13^C label (only m + 3 and m + 6 glucose were produced, Fig. 1*A*). Unlike glycerol, however, pyruvate/lactate and glutamine are likely to lose ^13^C label in the TCA cycle, potentially underestimating their contributions to glucose synthesis. All GNG substrates label the TPhos pool, consisting of GAP and DHAP in presumed rapid equilibrium—an assumption needed to perform this analysis. To explore further the labeling pattern of glucose and relative substrate use, we employed 2-^13^C–labeled glycerol, pyruvate, and lactate (the latter in a 1:10 ratio as above and indicated as ∗PL) in physiological fasting concentrations and repeated the mouse PHs study (Fig. 1, *E*–*H*). In this study, we continued to use U-^13^C glutamine to label glucose. Interestingly, the glucose labeling results were identical to those using U-^13^C–labeled substrates (compare Fig. 1, *A* and *E*), demonstrating in both cases more than 80% labeling of glucose by 2-^13^C glycerol and nearly 100% labeling of glucose when all labeled substrates were considered. We also found that physiological 2-^13^C glycerol predominantly labeled the triose phosphate pool (>70% of GAP labeled, Fig. 1*G*) *versus* minor labeling by either 2-^13^C pyruvate/lactate or U-^13^C glutamine. Therefore, low labeling of glucose by 2-^13^C pyruvate/lactate or U-^13^C glutamine in the unstimulated state reflects their intrinsically lower labeling of the TPhos pool, not loss of ^13^C label in the TCA cycle.

Study of TPhos pool labeling before and after glucagon stimulation was instructive when determining changes in substrate preference. Glucagon reduced the GAP-labeled fraction from 2-^13^C glycerol from 71% to 53%, maintained the GAP-labeled fraction from 2-^13^C pyruvate/lactate at ∼10%, and increased the GAP-labeled fraction from U-^13^C glutamine from 3% to 9% (Fig. 1*G*). These data suggest dilution of glycerol-labeled GAP by glutamine-labeled GAP. Consistent with these data, the m + 1/m + 2 ratio from 2-^13^C glycerol was also increased (*i.e.*, more of the TPhos pool is derived from glutamine and less from labeled glycerol after glucagon stimulation).

Finally, we used mass isotopomer distribution analysis (MIDA) to confirm the binomial relationship between the fractional-labeled product (glucose) and fractional concentration of both labeled substrates (DHAP and GAP). Many previous studies have shown that this relationship is valid even in the setting of unlabeled substrates (30). Originally, MIDA was used to estimate the fraction of glucose derived from new glucose synthesis in GNG (30). Here, we show that MIDA predicts the ^13^C fractional labeling pattern of glucose from fractional GAP labeling, assuming that GAP and DHAP are in rapid equilibrium and constitute 3-carbon monomers that are subsequently assembled as a 3-carbon dimer (compare Fig. 1, *F* and *H*). Moreover, the increase in the glucose m + 1/m + 2 ratio after glucagon treatment reflects a dilution of fractional GAP labeling by 2-^13^C glycerol with unlabeled 3-carbon substrate from glutamine.

### Intracellular mediators of glucagon action

**Figure 2:**
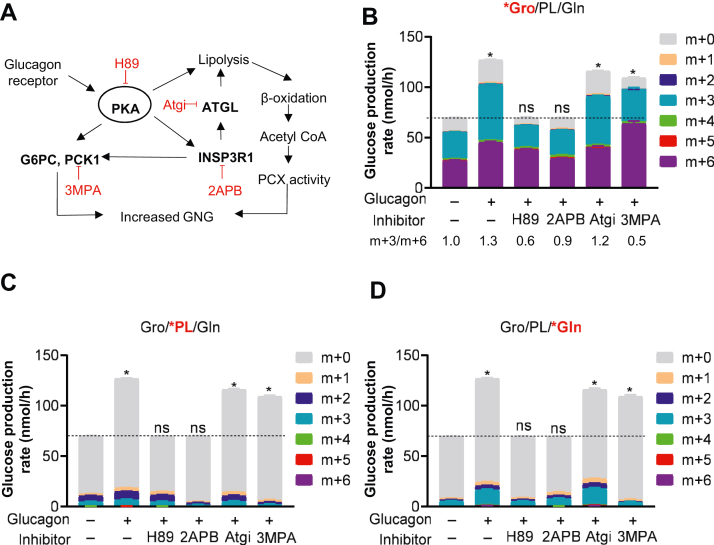
**Glucagon activates PKA and INSP3R1 for gluconeogenesis in primary hepatocytes.***A*, illustration of the mechanism underlying glucagon-stimulated GNG. Inhibitors are shown in *red*. *B*–*D*, glucose production and labeling pattern in mouse primary hepatocytes with treatment of 50 μM 2APB, 50 μM Atgi, 25 μM H89, or 500 μM 3MPA with/without 20 nM glucagon in the presence of four substrates labeled with ^13^C_3_ glycerol (*B*), ^13^C_3_ pyruvate/lactate (*C*), or ^13^C_5_ glutamine (*D*). ^13^C isotopologue glucose ratio (m + 3/m + 6) after ^13^C_3_ glycerol labeling is indicated in (*B*). All data are expressed as mean ± SEM., n = 6 biological replicates. ∗*p* < 0.01. Statistical analysis was performed using one-way ANOVA. All comparisons are against no-glucagon and no-inhibitor control groups. ^13^C isotopologues of glucose are indicated (m + 0 to m + 6). GNG, gluconeogenesis; INSP3R1, inositol triphosphate receptor 1; PCX, pyruvate carboxylase.

Glucagon is known to stimulate GNG through cAMP-mediated activation of PKA and increased transcription of GNG enzymes such as PCK1 and G6PC (Fig 2*A*) (11, 31). However, new evidence has emerged suggesting that glucagon could also function by increasing hepatic inositol triphosphate receptor 1 (INSP3R1) and adipose triglyceride lipase (ATGL) (8). Therefore, we employed inhibitors of PKA (H89) (32), INSP3R1 (2APB) (33), ATGL (Atgi) (34), and PCK1 (3MPA) (35) in the same labeling scheme as shown in Figure 1. Both PKA and INSP3R1 inhibitors blocked glucagon-mediated increases in glucose production in a concentration-dependent manner (Figs. 2, *B*–*D* and S2, *A* and *B*). In contrast, ATGL and PCK1 inhibitors only partially inhibited glucagon’s effect even at very high concentrations (Figs. 2, *B*–*D* and S2, *C* and *D*). Importantly, both PKA and INSP3R1 inhibitors (H89 and 2APB, respectively) neutralized the increased gluconeogenic flux from all three groups of substrates (Fig. 2, *B*–*D*). The ATGL inhibitor slightly suppressed flux from pyruvate/lactate but not from either glycerol or glutamine (Fig. 2, *B*–*D*). On the other hand, the PCK1 inhibitor (3MPA) clearly suppressed flux from both pyruvate/lactate and glutamine but not from glycerol (Fig. 2, *B*–*D*). Interestingly, however, 3MPA did alter the m + 3/m + 6 ratio after glycerol labeling (Fig. 1*B*) from 1.3 to 0.5, indicating less unlabeled TCA-derived substrates supplied the other 3-carbon molecule to glucose.

### A concentration-dependent increase in gluconeogenic enzyme expression by glucagon

**Figure 3:**
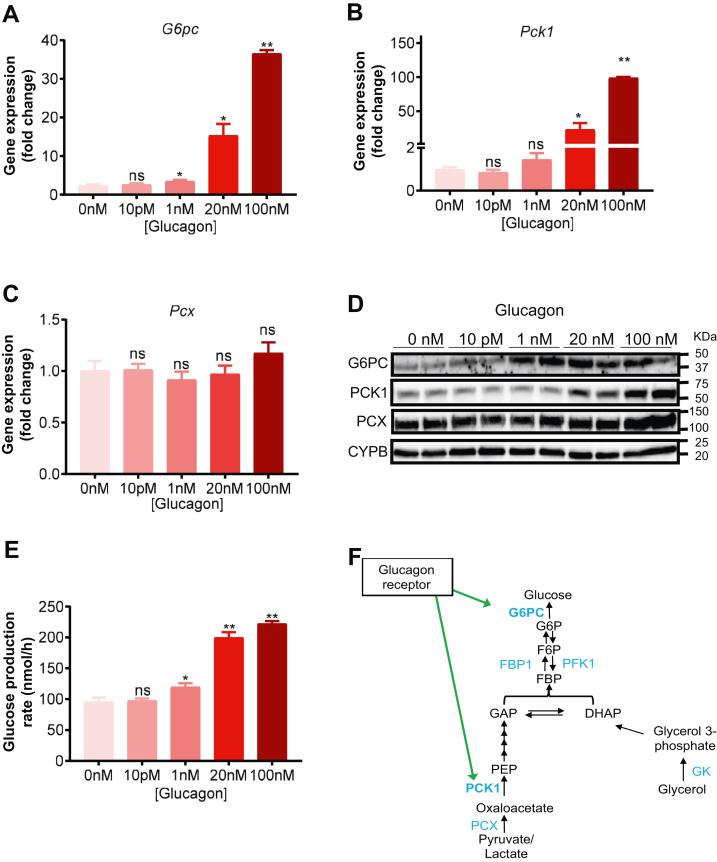
**Glucagon-induced gluconeogenesis strongly correlates with changes in enzyme expression.** mRNA levels (normalized to *Actb*) of *G6pc* (*A*), *Pck1* (*B*), and *Pcx* (*C*) in mouse primary hepatocytes treated with increasing concentrations of glucagon. n = 6 biological replicates per group. *D*, Western blot for G6PC, PCK1, and PCX proteins treated with increasing concentrations of glucagon. CYPB is a loading control. *E*, glucose production rate (nmol/h) in mouse primary hepatocytes treated with increasing concentrations of glucagon. n = 6 biological replicates per group. *F*, a model of glucagon action on the GNG pathway showing the stimulation of PCK1 and G6PC expression. All data are expressed as mean ± SEM. ∗∗*p* < 0.01; ∗*p* < 0.05; ns = not significant. Statistical analysis was performed using one-way ANOVA. All comparisons are against the no-glucagon and no-inhibitor control groups. CYPB, cyclophilin b; GNG, gluconeogenesis; PCX, pyruvate carboxylase.

To understand the roles of gluconeogenic enzymes in substrate utilization, we treated mouse PHs with various concentrations of glucagon and measured gluconeogenic enzyme expression (Fig. 3, *A*–*C*) and corresponding protein levels (Fig. 3*D*). *G6pc* expression correlates with the glucose production rate as both showed a significant increase at 1 nM glucagon (Fig. 3, *A*, *D*, and *E*). The expression of *Pck1* showed a similar trend, except that the increase at 1 nM concentration is not statistically different from control PHs (Fig. 3*B*). In contrast, PCX expression was not changed by glucagon treatment (Fig. 3*C*) as also reported (8). Contrary to a previous report, these data clearly show that glucagon’s stimulatory effect on gluconeogenic enzyme expression is a plausible mechanism for increased glucose production in PHs (Fig. 3*F*, ref. (8)).

### Forskolin recapitulates glucagon’s effect on substrate usage in GNG

**Figure 4:**
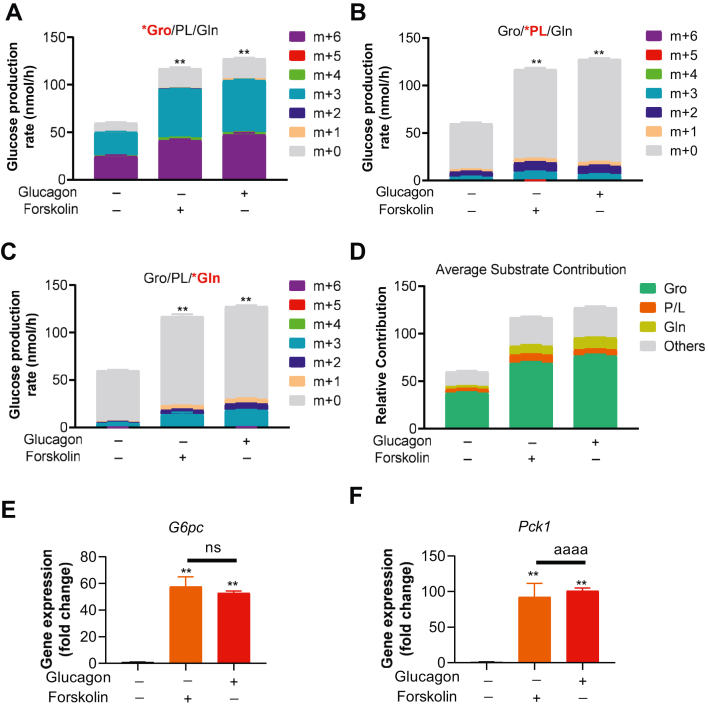
**Forskolin-induced PKA activation mimics glucagon’s effect.***A*, labeling pattern of glucose production in mouse pH treated with 10 μM forskolin or 20 nM glucagon in the presence of four substrates labeled with ^13^C_3_ glycerol (*A*), ^13^C_3_ pyruvate/lactate (*B*), or ^13^C_5_ glutamine (*C*). *D*, average substrate contribution to glucose production as shown in *A*–*C*. *E* and *F*, gene expression of *G6pc* (*E*) and *Pck1* (*F*) in PH treated with 10 μM forskolin or 20 nM glucagon in the presence of four substrates. All data are expressed as mean ± SEM. ∗∗*p* < 0.01; ns = not significant, n = 6 biological replicates. Statistical analysis was performed using one-way ANOVA. All comparisons are against no-glucagon and no-forskolin control groups, unless indicated by *solid line*.

Glucagon’s effect on PHs can be largely explained by increased *Pck1* and *G6pc* expression secondary to cAMP, calcium signaling, or both (Fig. 2*A*). Given our focus on determining substrate-specific gluconeogenic flux, we wanted to determine if glucagon’s effect on GNG could be reproduced in PHs using forskolin (a direct adenylyl cyclase activator). Using identical labeling schemes, glucagon and forskolin produced the same changes in glucose production and relative substrate utilization (Fig. 4, *A*–*D*) and expression of *G6pc* and *Pck1* (Fig. 4, *E* and *F*). These data indicate that forskolin-mediated PKA activation recapitulates glucagon-mediated regulation of substrates use in GNG.

### Hepatic PKA activation increases GNG *in vivo*

The findings on glucagon action presented so far were derived from *in vitro* studies in PHs. Some investigators have attempted to study the global action of glucagon *in vivo* using either acute injections or chronic infusions of glucagon (4). Systemic treatments, however, affect both the liver and peripheral tissues, such as adipose depots and the pancreas, making localization of glucagon’s effect problematic. Given that forskolin, a direct adenylyl cyclase activator, completely recapitulated glucagon’s effect in PHs (Fig. 4), we modeled elevated hepatic glucagon action by constitutive PKA activation using hepatic knockout of the PKA regulatory subunit in *Prkar1a*^fl/fl^ mice (36).

**Figure 5:**
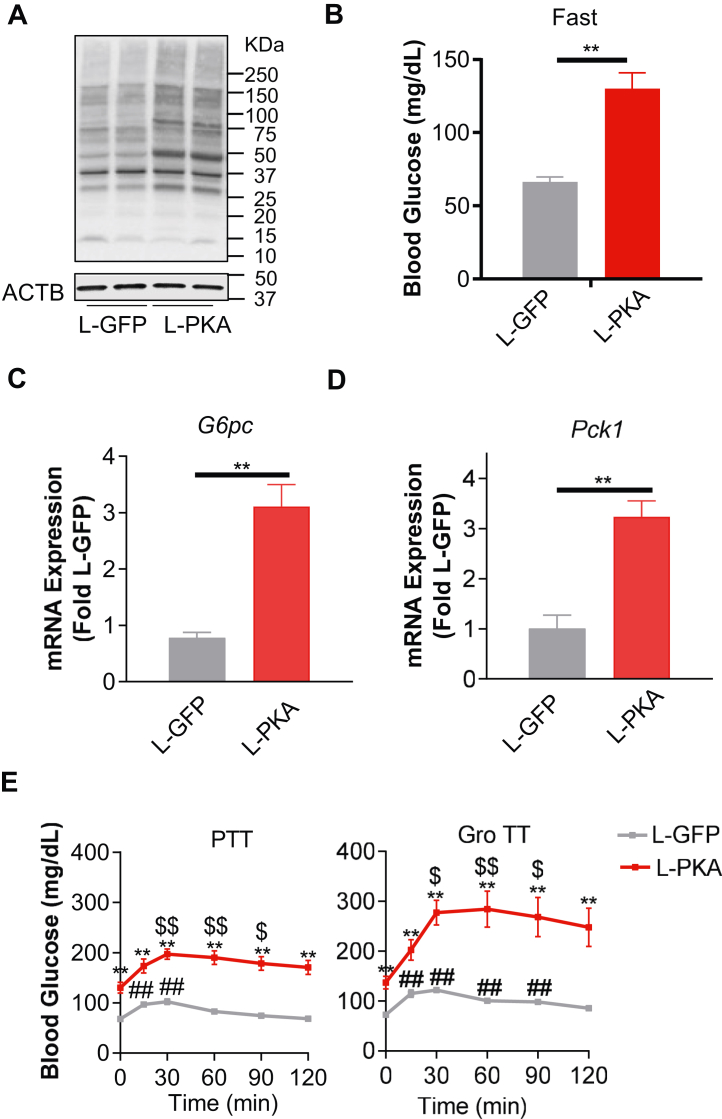
**PKA activation increases gluconeogenesis *in vivo*.***A*, Western blot of phospho-PKA substrates from liver samples fasted for 12 h. ACTB is a loading control. *B*, fasting glucose after fasting for 12 h. n = 6 to 8 per group. *C* and *D*, relative expression of *G6pc* (*C*) and *Pck1* (*D*) in the liver of the indicated groups after fasting for 12 h. Expression levels are normalized to ACTB and shown as fold change to L-GFP controls. n = 5 per group. *E*, pyruvate and glycerol tolerance test (PTT and Gro TT, respectively). Mice of the indicated groups were fasted for 12 h and then injected by i.p. with a bolus of glycerol (Gro TT; 9 mmol/kg) or sodium Pyr (PTT; 9 mmol/kg). n = 1012 per group. All data are expressed as mean ± SEM. ∗ denotes comparisons to the L-GFP group. ∗∗*p* < 0.01. $ denotes comparisons to the time 0, L-PKA group. ^$$^*p* < 0.01; ^$^*p* < 0.05. # denotes comparisons to the time 0, L-GFP group. ^##^*p* < 0.01; ^#^*p* < 0.05. Statistical analysis was performed using one-way ANOVA and post hoc testing in (*E*) and using *t*-tests in *B*–*D*. ACTB, β-actin.

Littermates of *Prkar1a*^fl/fl^ mice were placed on a normal chow for 16 weeks and then injected with either Ad-CRE or control Ad-GFP virus to generate experimental groups: L-GFP and L-PKA. Ad-CRE significantly reduced the expression of *Prkar1a* proteins in the liver to activate hepatic PKA without differential expression of *Prkar1a* proteins in other tissues such as skeletal muscle (Fig. S3*A*). L-GFP and L-PKA mice showed similar body weight (Fig. S3*B*) and fat mass (Fig. S3*C*). As expected, increased PKA activity was found in the liver of L-PKA mice compared to L-GFP control (Fig. 5*A*), resulting in increased fasting glucose (Fig. 5*B*) due to enhanced GNG in the liver. Consistent with findings in PHs, hepatic PKA activation in L-PKA mice significantly increased *G6pc* (Fig. 5*C*) and *Pck1* (Fig. 5*D*) expression compared to L-GFP mice. As expected, PKA activation (L-PKA) significantly increased glucose production from i.p. injection of either Pyr (pyruvate tolerance test, Fig. 5*E*) or Gro (glycerol tolerance test, Fig. 5*E*). Higher glucose levels found in L-PKA mice were positively correlated with serum insulin levels (Fig. S3*D*) and negatively correlated with serum glucagon levels (Fig. S3*E*).

### PKA activation affects lactate contribution to GNG *in vivo*

To investigate if PKA activation affects gluconeogenic flux *in vivo*, we infused L-GFP and L-PKA mice with ^13^C_3_ glycerol (Fig. S4*A*) or a mixture of ^13^C_3_ pyruvate/lactate (in a physiological ratio of 1:10) (Fig. S4*B*) following a 12-h fast. After a 6-h infusion of either tracer, all isotopologues of serum glucose reached steady state, and no significant changes in metabolite pool size were observed after the infusion (Fig. S4, *C* and *D*), indicating nonperturbative conditions were achieved. ^13^C enrichment after either glycerol or pyruvate/lactate infusion was also similar (∼15%) among the groups. The endogenous turnover rate (F_circ_) of circulating glycerol and lactate was calculated as previously described (24, 27).

**Figure 6:**
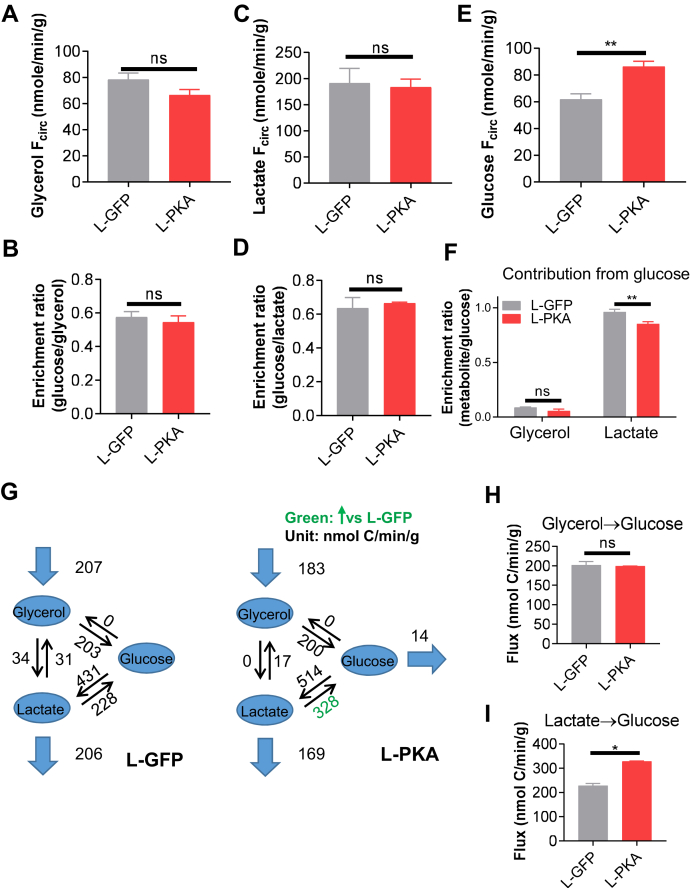
**PKA activation affects lactate contribution to GNG *in vivo*.***A* and *B*, nonperturbative infusion of ^13^C_3_ glycerol showing the turnover rate (F_circ_) of circulatory glycerol (*A*) and glucose enrichment ratio (*B*). *C* and *D*, nonperturbative infusion of ^13^C_3_ pyruvate/lactate showing the turnover rate (F_circ_) of circulatory lactate (*C*) and glucose enrichment ratio (*D*). *E*, nonperturbative infusion of ^13^C_3_ glucose showing the turnover rate (F_circ_) of circulating glucose. *F*, enrichment ratio of glycerol and lactate from glucose. *G*, *in vivo* gluconeogenic flux analysis. Best-fit flux values (μmol C/min) are shown. Fluxes that significantly increased or decreased against L-GFP groups are indicated in *green* and *red colors*, respectively. *H* and *I*, best-fit flux values with 95% confidence intervals for gluconeogenic flux from glycerol (*H*) and lactate (*I*). Data are expressed as best-fit value ± 95% flux confidence intervals based on calculations in (*G*). All data are expressed as mean ± SEM. ∗∗*p* < 0.01; ∗*p* < 0.05; ns = not significant. Statistical analyses were performed using *t* test. All comparisons are against L-GFP. GNG, gluconeogenesis; ns, non significant.

Both L-GFP and L-PKA mice showed a similar glycerol turnover rate (Fig. 6*A*), indicating similar glycerol production and consumption. Because glycerol is the only ^13^C source at steady-state conditions, its fractional contribution to glucose can be estimated based on a ratio of the average ^13^C enrichment (Fig. 6*B*). We previously reported that glycerol contributed the majority of the glucose carbon in normal fasted mice, both as a direct hepatic substrate and by labeling lactate in the Cori cycle (24). We now show that glycerol also contributes approximately 60% to glucose carbon in both L-GFP and L-PKA (Fig. 6*B*). Given that PHs do not generate m + 1 or m + 2 glucose labeling after the treatment with ^13^C_3_ glycerol, m + 1 and m + 2 serum glucose found after *in vivo* glycerol labeling (Fig. S4*A*) most likely represent glucose synthesized from labeled lactate generated from a glycerol→glucose→lactate pathway that undergoes ^13^C carbon loss in the TCA cycle.

The lactate turnover rate was also similar in both groups (Fig. 6*C*), and pyruvate/lactate contributed ∼65% of glucose carbon in L-GFP and L-PKA mice (Fig. 6*D*). It should be noted that both glycerol and pyruvate/lactate each contributed more than 50% to glucose carbon because a significant portion of pyruvate/lactate carbon originates from glycerol at steady state (Fig. S4*E*) (24). In contrast, <10% of glycerol carbon originated from pyruvate/lactate (Fig. S4*F*), indicating that the net flow of carbon is from glycerol to pyruvate/lactate. We also measured the glucose turnover rate (Fig. 6*E*) and its contribution to circulating glycerol and lactate (Fig. 6*F*) by infusing mice with ^13^C_6_-glucose. In contrast to glycerol and lactate turnover, L-PKA mice showed a significantly increased glucose turnover rate compared to the L-GFP control (Fig. 6*E*). Glucose contributed more than 85% of lactate carbon but only 10% of glycerol carbon in both groups (Fig. 6*F*), consistent with a predominant glycerol→glucose→lactate carbon flow.

To illustrate more clearly how the *in vivo* carbon flows among glycerol, lactate, and glucose, we performed a metabolic flux analysis using the enrichment and F_circ_ data and a previously developed model (Fig. 6*G* and ref. (24)). L-PKA mice showed a significant increase in gluconeogenic flux from lactate compared to L-GFP mice (Fig. 6*I*), while gluconeogenic flux from glycerol showed a similar flux (Fig. 6*H*).

## Discussion

GNG has been extensively studied by a number of investigators. While other studies have linked both glutamine and pyruvate/lactate metabolism to glucagon’s action in PHs (8, 20), these studies were performed at superphysiological fasting concentrations and failed to consider glycerol’s role as a primary substrate. They must now be interpreted with caution, given that glycerol is the primary substrate used by PHs in the absence or presence of glucagon. In particular, glucagon stimulation of PHs markedly increased glycerol production (8), suggesting that it is necessary to separate glucose produced by pyruvate/lactate due to lipolysis (FA oxidation→acetyl-CoA→PCX activation, Fig. 2*A*) from glucose produced from glycerol liberated during lipolysis.

Under basal conditions, PHs ^13^C enrichment data indicate that nonglycerol substrates are minor substrates compared to glycerol. We rigorously considered the hypothesis that ^13^C carbon loss from all nonglycerol substrates in the TCA cycle minimized their glucose labeling contributions. Ultimately, we rejected this hypothesis because both the metabolic flux analysis (U-^13^C–labeled substrates) and MIDA (2-^13^C–labeled substrates) studies demonstrate that glycerol is the primary substrate labeling glucose (Fig. 1, *D* and *F*) or the TPhos pool metabolites (Figs. 1*G* and S1*C*) under physiologic fasting conditions.

After glucagon stimulation, PH ^13^C enrichment data demonstrate increased use of all substrates, but glutamine use is preferentially increased (Fig. 1, *D* and *F*). We also considered if ^13^C carbon loss from pyruvate/lactate minimizes its glucose labeling contribution, but we rejected this hypothesis for the following two reasons: (1) Glutamine’s but not pyruvate/lactate’s contribution to glucose carbon increased after glucagon stimulation, even though both enter the TCA cycle (Fig. 1, *C* and *D*) and (2) ^13^C enrichment of TCA intermediates by glutamine but not pyruvate/lactate increases after glucagon (Fig. S1*C*). Consistent with the latter finding, Miller *et al.* (20) showed that glucagon increased glutamine flux in GNG through the activation of α-ketoglutarate dehydrogenase. We conclude that our results reflect the relative activity of these GNG pathways in mouse PHs toward different substrates depending on their concentration and glucagon stimulation.

After depleting tissue glycogen, carbon reservoirs for GNG include gluconeogenic AAs from proteolysis or glycerol from lipolysis. Among all gluconeogenic substrates, lactate, pyruvate, alanine, glutamine, and glycerol together contribute more than 97% of all carbons to glucose (37). Lactate and alanine are first converted to pyruvate and then to oxaloacetate by PCX. Attention at this GNG entry point is warranted given previous studies and very high lactate flux in mammals (27). However, high lactate flux does not necessarily mean high net carbon flow to glucose, given that a significant amount of lactate is fully metabolized to CO_2_ (27). Our data extend this finding to show that glycerol is the ultimate carbon source for more than 60% of circulating lactate and glucose in mice with or without activation of the PKA signaling pathway by glucagon. Thus, glycerol can enter GNG directly or after conversion to lactate *via* glycerol→glucose→lactate metabolism in the Cori cycle. Fasting hypoglycemia, hyperketonemia, and developmental delay in patients with defects in glycerol metabolism also suggest that glycerol is essential for GNG in humans (38). Finally, glycerol’s importance as a major substrate takes on even greater importance in obese patients with increased circulating glycerol levels (39, 40).

## Experimental procedures

### Primary hepatocyte isolation

Age-matched C57BL/6J albino males between 3 and 4 months were used for PH culture isolation. All mice were anesthetized using a ketamine/xylazine mixture (Henry Schein) before isolation. The hepatic portal vein was cannulated and perfused with Kreb’s Ringer Bicarbonate Buffer (Sigma-Aldrich) containing EGTA for 10 min at 37 °C. After the first wash, a second Kreb’s Ringer wash containing CaCl_2_ and Liberase (Roche Holding AG) was applied for 10 min at 37 °C. Hepatocytes were filtered through a gauze mesh and resuspended in plating media: William’s Media E (Sigma-Aldrich) containing 10% fetal bovine serum (Sigma-Aldrich), 200 nM dexamethasone (Sigma-Aldrich), 1 × penicillin/streptomycin (Thermo Fisher Scientific), and 2 mM L-glutamine (Thermo Fisher Scientific). Cells were plated at a density of 3 × 10^5^/ml on a six-well collagen (Sigma-Aldrich)-coated plate. Hepatocytes were allowed to recover overnight and all experiments were started 24 h post isolation, unless otherwise indicated.

### *In vitro* isotope labeling experiments

After recovery at 37 °C and 5% CO_2_ from isolation and viral treatment, if applicable, PH were serum-starved for 3 h in a basal medium (no-glucose Dulbecco's modified Eagle's medium supplemented with 3.52 mg/ml Hepes, 1 × penicillin/streptomycin, and 2 mM L-Gln). For *in vitro* glucose production, the cells were washed once with 1 × PBS and treated with glucose production medium (no-glucose Dulbecco's modified Eagle's medium with 3.52 mg/ml Hepes and 1 × penicillin/streptomycin) and substrates with or without 20 nM glucagon. For the physiological-concentration substrate scheme, 0.5 mM L-glutamine, 0.33 mM glycerol, 0.25 mM sodium pyruvate, and/or 2.5 mM sodium lactate were used. For the high-concentration substrate scheme, 5 mM glycerol and 50 mM sodium lactate were used. Culture medium was collected every 2 h for the measurement of glucose production and labeling patterns. To compensate the loss of substrates and maintain the physiological concentration, 0.22 μmol glycerol, 0.33 μmol pyruvate, and 0.05 μmol L-glutamine per ml culture were added every 2 h after the sampling of media.

For the inhibition of PKA or PCK1, 25 μM H-89 dichloroacetate hydrate (Sigma-Aldrich) or 500 μM 3-Mercaptopropionic acid (3-MPA, Sigma-Aldrich) was directly dissolved in the glucose production medium. For the inhibition of INSP3R1 or ATGL, 50 μM 2APB (R&D Systems) or 50 μM atglistatin (Sigma-Aldrich) was first dissolved in the vehicle of 0.5% dimethyl sulfoxide or 0.1% ethanol, respectively, and then added to the glucose production medium. The total amount of glucose in the media was measured with the Glucose Assay Kit (Abcam, Cat# ab65333).

For ^13^C-labeled substrate experiments, the same conditions were used, but substrates were substituted one at a time with ^13^C_3_-sodium pyruvate/lactate, ^13^C_5_-L-glutamine, or ^13^C_3_-glycerol (Cambridge Isotope Lab, Inc) as indicated. The medium metabolites were directly extracted in 100 volumes of ice-cold 40:40:20 methanol:acetonitrile:water solution with 0.1% formic acid. The cells were washed once with prewarmed 1 × PBS and extracted with ice-cold 40:40:20 methanol:acetonitrile:water solution with 0.1% formic acid (2 ml per million cells). Both media and cell extracts were incubated on ice for 5 min and neutralized with 0.7% NH_4_HCO_3_, followed by centrifugation for 10 min at 16,000*g*. The supernatant was then transferred to another clean tube for mass spectrometry (LC-MS) analysis (41).

### Animals and animal care

The glucose homeostasis varies throughout the estrus cycle in female mice (42). To avoid the variation caused by estrus cycles, only male mice were used in this study. All mice were maintained on a C57BL/6J-albino background (Jackson Laboratory; B6(Cg)-Tyrc-2J/J). Littermates of Prkar1a^fl/fl^ mice (Jackson Laboratory; Prkaa1^tm1.1Sjm^/J) were placed on a normal chow for 11 weeks after weaning and then injected with either Ad5-CMV-CRE or Ad5-CMV-GFP (3 × 10^9^ pfu/mouse; University of Iowa Viral Vector Core) *via* the tail vein, resulting in L-GFP and L-PKA mice. All experiments were performed between 2 and 5 weeks after the injection. All mice described were housed in a pathogen-free barrier facility with a 12-h light/dark cycle. All animal protocols were approved by the Institutional Animal Care and Use Committee of Rutgers University.

### Protein extraction and Western blotting

Protein preparations were conducted with both hepatocytes that were lysed in Laemmli buffer with β-mercaptoethanol and tissue samples. For tissue protein preparations, the tissues had been snap-frozen in liquid nitrogen following the sacrifice of the animal and were mechanically homogenized in ice-cold RIPA buffer (Sigma-Aldrich) with 1 × protease inhibitor (Roche Holding AG) and 1 × phosphatase inhibitor (Thermo Fisher Scientific) using Bullet Blender (Next Advance, Inc). The homogenate was then centrifuged at 16,000*g* at 4 °C and the supernatant was collected. Protein concentrations were measured using the Pierce BCA assay (Thermo Fisher Scientific). Western blotting was performed using standard procedures utilizing the standard V3 Western Workflow (Bio-Rad Laboratories). All standard Western blotting reagents were obtained from Bio-Rad Laboratories. Antibodies were obtained from Cell Signaling Technology (CYPB (1:1000; Cat# 43603S), phospho-PKA substrate (1:1000; Cat# 9624S), anti-rabbit IgG, HRP-linked (1:2000; Cat# 7074S), anti-mouse IgG, HRP-linked (1:2000, Cat# 58802)), Abcam (PCK1 (1:1000; Cat# ab70358), PCX (1:1000; Cat# ab126707)), or Atlas (G6PC (1:250; Cat# HPA052324)).

### Metabolic tests

For the pyruvate and glycerol tolerance tests, mice were fasted overnight (12 h) and then injected i.p. with sodium pyruvate (9 mmol/kg; Sigma-Aldrich) or glycerol (9 mmol/kg; Sigma-Aldrich). Blood glucose measurements were obtained *via* a small nick in the lateral tail vein using a glucometer (Bayer Contour).

### Quantitative RT-PCR analysis

Total RNA was isolated from PHs and mouse livers using the TriZol method. Complementary DNA was obtained using iScript (Bio-Rad) and then subjected to quantitative RT-PCR analysis using SYBR Green (Bio-Rad) according to manufacturer’s protocol. The primers used for the analysis were the following: G6pc Forward 5′-CAGCAAGGTAGATCCGGGA-3′ Reverse 5′-AAAAAGCCAACGT ATGGATTCCG-3′; Pck1 Forward 5′-AGCATTCAACGCCAGGTTC-3′ Reverse 5′- CGAGTCTGTCAGTTCAATACCAA-3′; Pcx Forward 5′-TGGGTTCCTCTCAGAGCGAG-3′ Reverse 5′-GTCTCCCATCTTGCGGACC-3′; Gk Forward 5′-ATCCGCTGGCTAAGAGACAACC-3′ Reverse 5′-TGCACTGGGCTCCCAATAAGG-3′, Hnf4α Forward 5′-CTTCCTTCTTCATGCCAG-3′ Reverse 5′-ACACGTCCCCATCTGAAG-3′, Actb Forward 5′-CCAGTTGGTAACAATGCCATG-3′ Reverse 5′-GGCTGTATTCCCCTCCATCG-3′.

### Insulin, glycerol, and glucagon measurements

Serum insulin levels were measured using the Ultra-Sensitive Mouse Insulin ELISA Kit (Crystal Chem). Serum glycerol levels were measured using the Glycerol Assay Kit (Sigma-Aldrich). Serum glucagon levels were measured using the Glucagon ELISA Kit (Crystal Chem). Liver glycerol levels were calculated by subtracting the endogenous glycerol-3-phosphate level (measured using the Sigma Glycerol Assay Kit without the addition of ATP) from the sum of glycerol-3-phosphate and liver glycerol (measured using the Sigma Glycerol Assay Kit with the addition of ATP).

### *In vivo* isotope labeling studies

For continuous infusion experiments, five mice from each group (L-GFP and L-PKA) were catheterized on the right jugular vein (24, 43) and recovered more than 7 days. Catheterized mice were fasted for 12 h and then transferred to new cages without food and infused to circulating isotope steady state (5–6 h). For the mouse infusion, a tether and swivel system was used to allow mice for free movement in the cage (Instech Laboratories, Inc). Water-soluble isotope-labeled metabolite tracers (Cambridge Isotope Laboratories, Inc) were prepared as solutions in sterile normal saline and infused *via* the catheter at a constant rate (0.1 μl/g body weight/min). 200 mM U-^13^C glucose, 150 mM U-^13^C glycerol, or 40 mM U-^13^C sodium pyruvate with 360 mM U-^13^C lactate were infused. About 30 μl blood was collected by tail vein bleeding at each time point, left at room temperature in the absence of anticoagulant for 30 min, and centrifuged at 4 °C to prepare serum. Serum and tissue samples were kept at −80 °C until further extraction.

Serum (10 μl each) was mixed with the extraction solution (ice cold 40:40:20 methanol:acetonitrile:water solution with 0.1% formic acid), followed by vortexing for 10 s, incubation at 4 °C for 10 min, and centrifugation at 4 °C and 16,000*g* for 10 min. The volume of the extraction solution (in μl) was 25 × the volume of serum. The supernatant was transferred to a clean tube and neutralized with a 0.7% NH_4_HCO_3_ solution. The mixture was centrifuged again at 4 °C at 16,000*g* for 10 min. The supernatant was then transferred to another clean tube for mass spectrometry (LC-MS) analysis.

### Glycerol derivatization for LC-MS analysis

Due to poor ionization of glycerol, a derivatization reaction is required to detect glycerol in LC-MS. Samples containing glycerol were added into 10 × volume of reaction buffer containing 25 mM Tris (pH 8.0), 10 mM Mg^2+^, 50 mM NaCl, 5 mM ATP, and 2 U/ml glycerol kinase and incubated for 10 min at room temperature. The Gro ion counts equal the difference of glycerol-3-phosphate ion counts before and after the reaction.

### LC-MS analysis

LC conditions were optimized on an HPLC-ESIMS system fitted with a Dionex UltiMate 3000 HPLC and a Thermo Q Exactive Plus MS. The HPLC was fitted with a Waters XBridge BEH Amide column (2.1 mm × 150 mm, 2.5 μm particle size, 130 Å pore size) coupled with a Waters XBridge BEH XP VanGuard cartridge (2.1 mm × 5 mm, 2.5 μm particle size, 130 Å pore size) guard column. The column over temperature was set to 25 °C. The solvent A consisted of water/acetonitrile (95:5, V/V) with 20 mM NH_4_Ac and 20 mM NH_4_OH at pH 9. The solvent B consisted of acetonitrile/water (80:20, V/V) with 20 mM NH_4_Ac and 20 mM NH_4_OH at pH 9 in the following solvent B percentages over time: 0 min, 100%; 3 min, 100%; 3.2 min, 90%; 6.2 min, 90%; 6.5 min, 80%; 10.5 min, 80%; 10.7 min, 70%; 13.5 min, 70%; 13.7 min, 45%; 16 min, 45%; and 16.5 min, 100%. The flow rate was set to 300 μl/min, with an injection volume of 5 μl. The column temperature was set at 25 °C. Mass spectrometry scans were obtained in negative ion mode with a resolution of 70,000 at m/z 200, in addition to an automatic gain control target of 3 × 10^6^ and m/z scan range of 72 to 1000. Metabolite data was obtained using the MAVEN software package with each labeled isotope fraction (mass accuracy window: 5 ppm). The isotope natural abundance and tracer isotopic impurity were corrected using AccuCor (44).

### Flux analysis

For *in vitro* experiments, the substrate-specific GNG fluxes were calculated using an elementary mass unit method as previously described (17). This model reproducibly predicts flux for glycerol, pyruvate/lactate, and glutamine. For *in vivo* experiments, the turnover rates of circulating glycerol, lactate, and glucose were calculated using the following equation (28):

$$F_{circ}=Infusion\;rate*\left(\frac{1}{fraction\;of\;tracer}-1\right)$$

*In vivo* fluxes were optimized to fit the enrichment of circulating glucose, lactate, and glycerol using the R package DEoptim. The 95% confidence intervals were calculated as previously described (45). Code for *in vivo* flux analysis is available at: https://github.com/wangyujue23/PKA-manuscript.

### Statistical analysis

All analysis and graphs were done on GraphPad Prism 7.0 software (https://www.graphpad.com/scientific-software/prism/). Statistics were performed using either Student’s *t* test or one-way ANOVA where appropriate.

## Data availability

The data are available within the article and supplemental information.

## Supporting information

This article contains supporting information.

## Conflict of interest

The authors declare no competing interests.

## Supporting information

Figure S1 **Substrate labeling and flux of gluconeogenesis intermediates.***A*, glucose production rate in primary hepatocytes during 8 h culture with individual or all four substrates at physiologic fasting concentration (0.33 mM glycerol, 0.25 mM pyruvate/2.5 mM lactate (PL) or 0.5 mM L-glutamine) and treated with or without 20 nM glucagon. *B*, constant media substrate concentrations during glucose production assay maintained by supplementation. *C*, average ^13^C enrichment of gluconeogenic intermediates with or without treatment of 20 nM glucagon in primary hepatocytes with all four substrates (0.33 mM glycerol, 0.5 mM L-glutamine and 0.25 mM/2.5 mM pyruvate/lactate) labeled with U-^13^C one at a time. *D*, glucose production rate in mouse primary hepatocytes treated with 20 nM glucagon, or 0.2 mM free fatty acids (FFA, 200 μm palmitate and 200 μm oleate conjugated to 2% BSA), or both in the presence of four substrates labeled with ^13^C_3_ glycerol, ^13^C_3_pyruvate/lactate or ^13^C_5_ glutamine. *E*, total ion count of cellular acetyl-CoA in primary hepatocytes treated with 20 nM glucagon, or 0.2 mM free fatty acids, or both in the presence of four substrates labeled with ^13^C_3_ glycerol, ^13^C_3_ pyruvate/lactate or ^13^C_5_ glutamine. All data are expressed as mean ± SEM. ∗∗*p* < 0.01; ∗*p* < 0.05; ns = not significant. Statistical analysis was performed using ANOVA testing between glucagon-treated and control group. n = 6 biological replicates.

Figure S2 **Inhibition of the glucagon-stimulatory pathway.***A*–*D*, *in vitro* glucose production rate in isolated hepatocytes treated with 20 nM glucagon and H89 (*A*), 2APB (*B*), Atgi (*C*) or 3MPA (*D*) over a range of concentrations. All data are expressed as mean ± SEM. *Dashed line* indicates the base line glucose production rate without glucagon. ∗ denotes the comparison between no-glucagon and no-inhibitor control group. ∗∗*p* < 0.01; ∗*p* < 0.05. $ denotes the comparisons to glucagon treatment without inhibitors. ^$$^*p* < 0.01; ^$^*p* < 0.05; ns = not significant. Statistical analysis was performed using one-way ANOVA. n = 6 biological replicates per group.

Figure S3 **PKA activation in hepatic PRKAR1α KO mice.***A*, Western blot showing the protein level of PRKAR1α in the liver and skeletal muscle after the indicated viral injection. *B*, body weights of indicated two groups after 12 h fast. *C*, epididymal fat mass of indicated two groups after 12 h fast. *D*, Serum insulin level of indicated two groups following a 12-h fast. *F*, serum glucagon level of indicated two groups following a 12-h fast. All data are expressed as mean ± SEM. ∗∗*p* < 0.01; ∗*p* < 0.05; ns = not significant. Statistical analyses were performed using t-test. All comparisons are against L-GFP. n = 6 to 8 per group.

Figure S4 **Time course of tracer infusions.***A* and *B*, labeled fraction of circulating glucose at indicated time points showing that glucose enrichment reached steady state after 6-h infusion. Indicated two groups of mice were infused with ^13^C_3_ glycerol (*A*) or ^13^C_3_pyruvate/lactate (*B*) for 6 h after 12 h fast. *C* and *D*, pool size of glycerol (*C*) and lactate (*D*) before and after 6 h infusion in indicated groups. *E* and *F*, ^13^C enrichment of pyruvate, lactate, glycerol, and glucose after a 6 h infusion of ^13^C_3_ glycerol (*E*) or ^13^C_3_ pyruvate/lactate (*F*). All data are expressed as mean ± SEM. ns = not significant. Statistical analyses were performed using multiple t-test. n = 4 to 5 per group. ^13^C isotopologues of glucose are indicated (m + 0 to m + 6)
