# Supplementary figures and images for: Glucagon changes substrate preference in gluconeogenesis
Source: J Biol Chem. 2022 Nov 17;298(12):102708. doi: 10.1016/j.jbc.2022.102708 (PMC9747632; doi:10.1016/j.jbc.2022.102708)

Figure S1

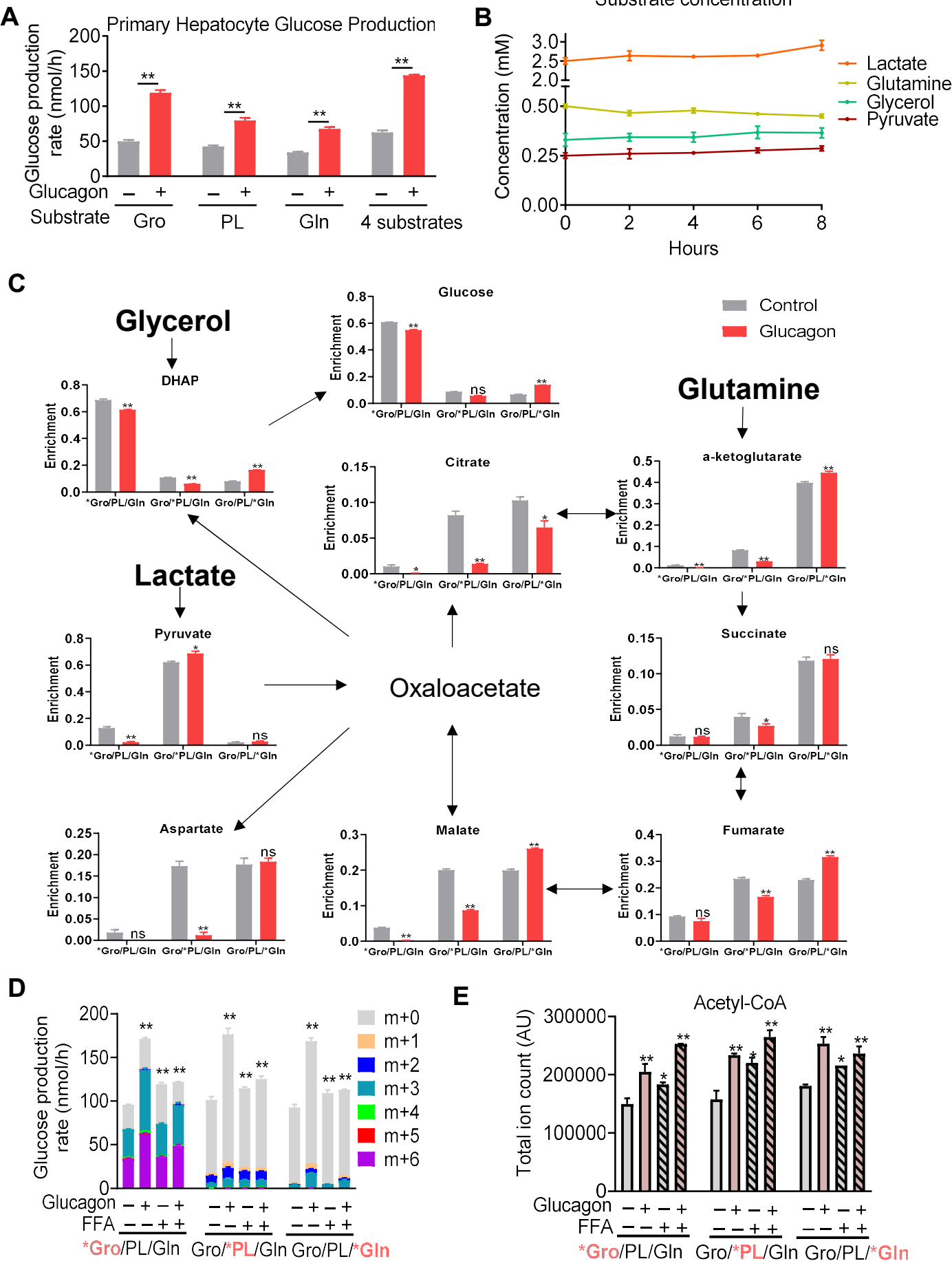

Supplement: Figure S1 — Substrate labeling and flux of gluconeogenesis intermediates.A, glucose production rate in primary hepatocytes during 8 h culture with individual or all four substrates at physiologic fasting concentration (0.33 mM glycerol, 0.25 mM pyruvate/2.5 mM lactate (PL) or 0.5 mM L-glutamine) and treated with or without 20 nM glucagon. B, constant media substrate concentrations during glucose production assay maintained by supplementation. C, average 13C enrichment of gluconeogenic intermediates with or without treatment of 20 nM glucagon in primary hepatocytes with all four substrates (0.33 mM glycerol, 0.5 mM L-glutamine and 0.25 mM/2.5 mM pyruvate/lactate) labeled with U-13C one at a time. D, glucose production rate in mouse primary hepatocytes treated with 20 nM glucagon, or 0.2 mM free fatty acids (FFA, 200 μm palmitate and 200 μm oleate conjugated to 2% BSA), or both in the presence of four substrates labeled with 13C3 glycerol, 13C3pyruvate/lactate or 13C5 glutamine. E, total ion count of cellular acetyl-CoA in primary hepatocytes treated with 20 nM glucagon, or 0.2 mM free fatty acids, or both in the presence of four substrates labeled with 13C3 glycerol, 13C3 pyruvate/lactate or 13C5 glutamine. All data are expressed as mean ± SEM. ∗∗p < 0.01; ∗p < 0.05; ns = not significant. Statistical analysis was performed using ANOVA testing between glucagon-treated and control group. n = 6 biological replicates. [file mmc1.pdf]

Figure S2

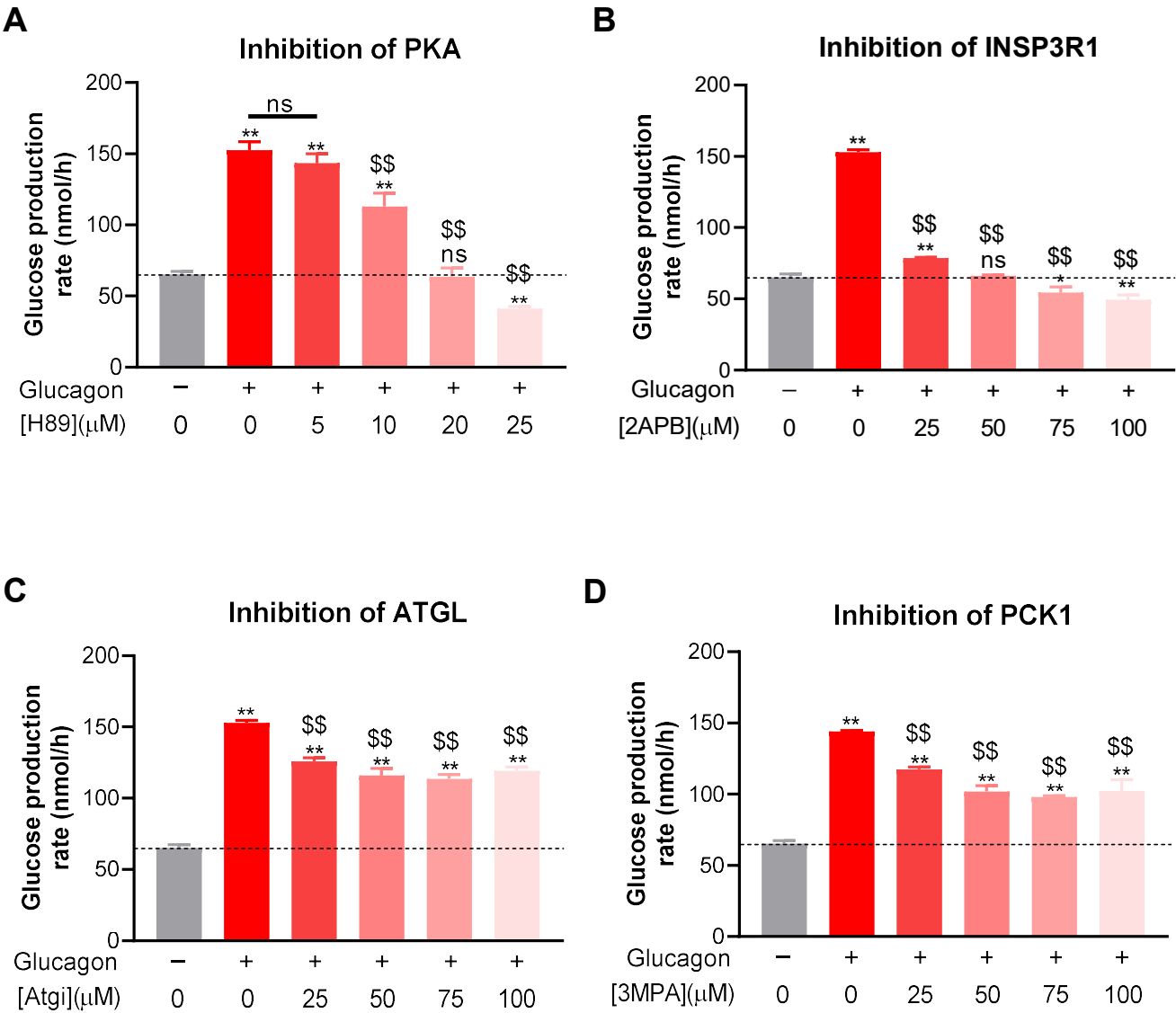

Supplement: Figure S2 — Inhibition of the glucagon-stimulatory pathway.A–D, in vitro glucose production rate in isolated hepatocytes treated with 20 nM glucagon and H89 (A), 2APB (B), Atgi (C) or 3MPA (D) over a range of concentrations. All data are expressed as mean ± SEM. Dashed line indicates the base line glucose production rate without glucagon. ∗ denotes the comparison between no-glucagon and no-inhibitor control group. ∗∗p < 0.01; ∗p < 0.05. $ denotes the comparisons to glucagon treatment without inhibitors. $$p < 0.01; $p < 0.05; ns = not significant. Statistical analysis was performed using one-way ANOVA. n = 6 biological replicates per group. [file mmc2.pdf]

Figure S3

**A**

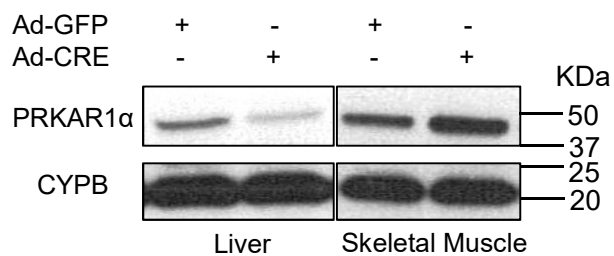

**B**

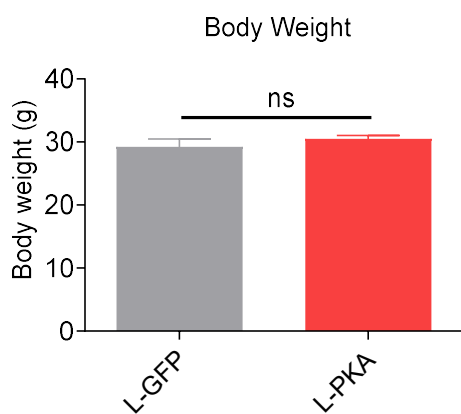

**C**

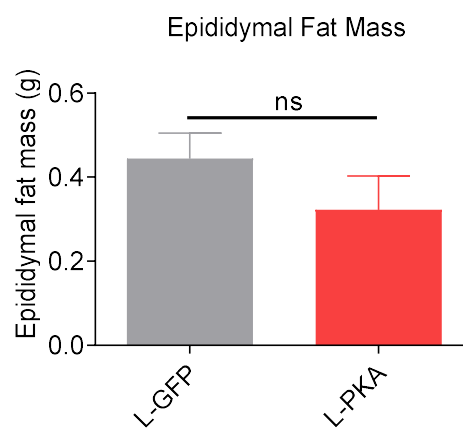

**D**

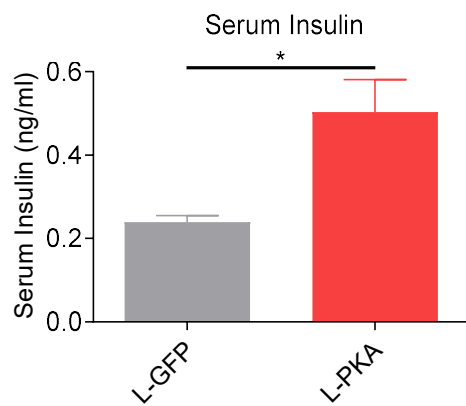

**E**

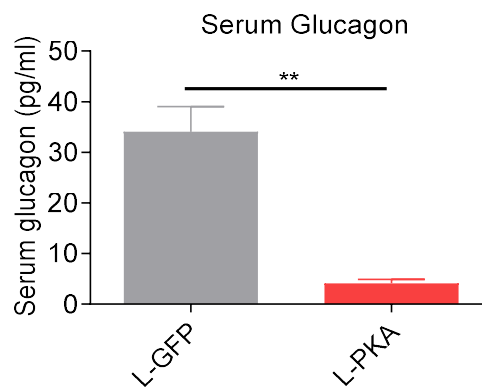

Supplement: Figure S3 — PKA activation in hepatic PRKAR1α KO mice.A, Western blot showing the protein level of PRKAR1α in the liver and skeletal muscle after the indicated viral injection. B, body weights of indicated two groups after 12 h fast. C, epididymal fat mass of indicated two groups after 12 h fast. D, Serum insulin level of indicated two groups following a 12-h fast. F, serum glucagon level of indicated two groups following a 12-h fast. All data are expressed as mean ± SEM. ∗∗p < 0.01; ∗p < 0.05; ns = not significant. Statistical analyses were performed using t-test. All comparisons are against L-GFP. n = 6 to 8 per group. [file mmc3.pdf]

Figures S4: Time course of tracer infusions

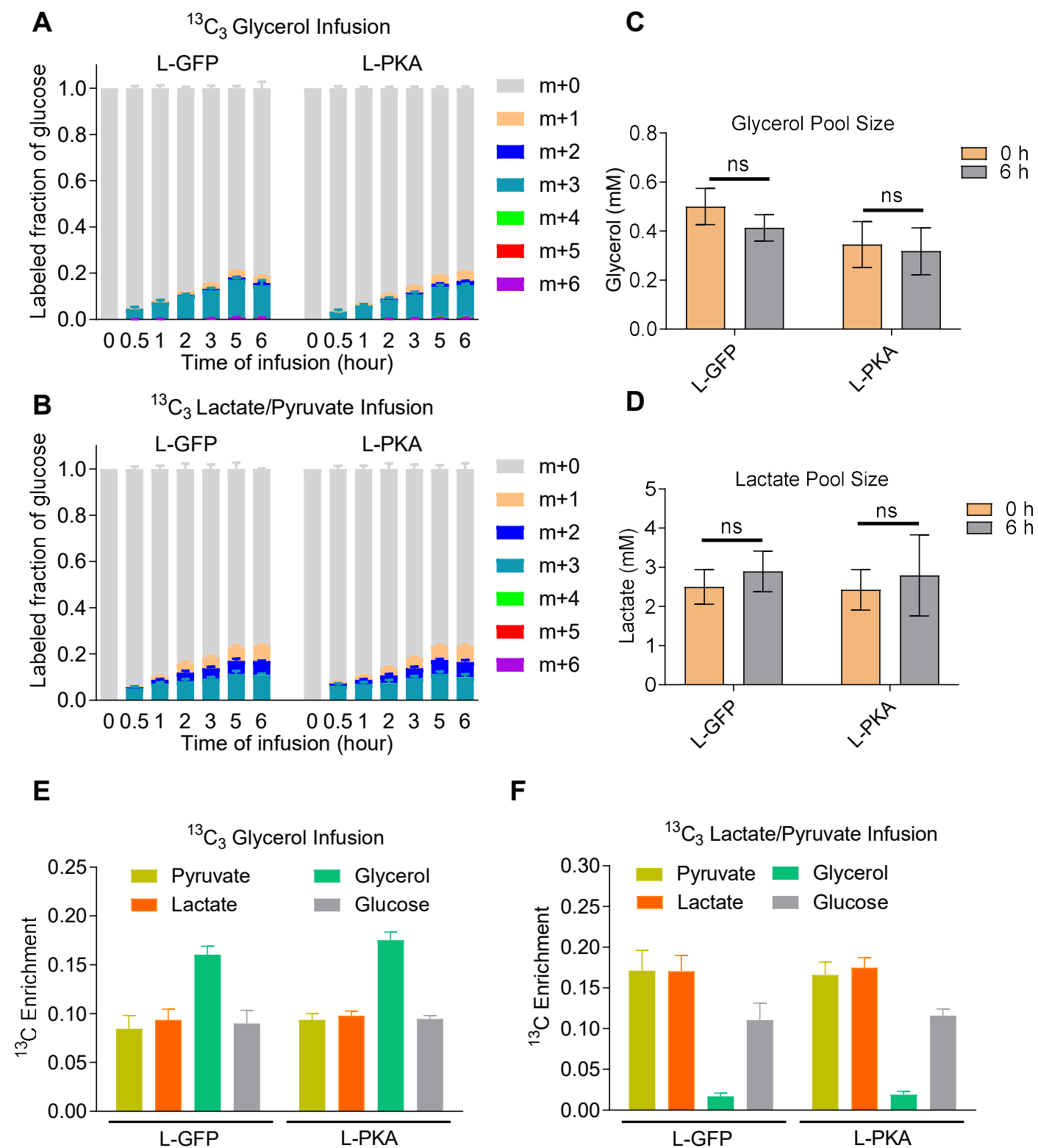

Supplement: Figure S4 — Time course of tracer infusions.A and B, labeled fraction of circulating glucose at indicated time points showing that glucose enrichment reached steady state after 6-h infusion. Indicated two groups of mice were infused with 13C3 glycerol (A) or 13C3pyruvate/lactate (B) for 6 h after 12 h fast. C and D, pool size of glycerol (C) and lactate (D) before and after 6 h infusion in indicated groups. E and F, 13C enrichment of pyruvate, lactate, glycerol, and glucose after a 6 h infusion of 13C3 glycerol (E) or 13C3 pyruvate/lactate (F). All data are expressed as mean ± SEM. ns = not significant. Statistical analyses were performed using multiple t-test. n = 4 to 5 per group. 13C isotopologues of glucose are indicated (m + 0 to m + 6) [file mmc4.pdf]
